# Interactions Between Therapeutics for Metabolic Disease, Cardiovascular Risk Factors, and Gut Microbiota

**DOI:** 10.3389/fcimb.2020.530160

**Published:** 2020-10-23

**Authors:** Qi-You Ding, Jia-Xing Tian, Min Li, Feng-Mei Lian, Lin-Hua Zhao, Xiu-Xiu Wei, Lin Han, Yu-Jiao Zheng, Ze-Zheng Gao, Hao-Yu Yang, Xin-Yi Fang, Xiao-lin Tong

**Affiliations:** ^1^Department of Endocrinology, Guang'anmen Hospital, China Academy of Chinese Medical Sciences, Beijing, China; ^2^Graduate College, Beijing University of Traditional Chinese Medicine, Beijing, China

**Keywords:** metabolic therapy, gut microbiota, cardiovascular diseases, metformin, microbiota-targeted therapies

## Abstract

With improved standards of living, the incidence of multiple metabolic disorders has increased year by year, especially major risk factors for cardiovascular disease such as hyperglycemia and hyperlipidemia, continues to increase. Emerging epidemiological data and clinical trials have shown the additional protective effects of some metabolic therapy drugs against cardiovascular diseases. A series of studies have found that these drugs may work by modulating the composition of gut microbiota. In this review, we provide a brief overview of the contribution of the gut microbiota to both metabolic disorders and cardiovascular diseases, as well as the response of gut microbiota to metabolic therapy drugs with cardiovascular benefits. In this manner, we link the recent advances in microbiome studies on metabolic treatment drugs with their cardiovascular protective effects, suggesting that intestinal microorganisms may play a potential role in reducing cardiovascular risk factors. We also discuss the potential of microorganism-targeted therapeutics as treatment strategies for preventing and/or treating cardiovascular disease and highlight the need to establish causal links between therapeutics for metabolic diseases, gut microbiota modulation, and cardiovascular protection.

## Introduction

Human dietary habits and lifestyle have changed greatly over time with the development of society (Chauveau et al., 2013). At the same time, the number of people suffering from metabolic diseases such as type 2 diabetes mellitus (T2DM), obesity, and dyslipidemia has also grown rapidly (Whiting et al., 2011; Loh et al., 2019). These metabolic disorders have proven to be major risk factors for cardiovascular diseases (CVD) (DeFronzo and Ferrannini, 1991; Barr et al., 2007). Despite adherence to recommended therapies, especially multi-drug combination therapy for patients with multiple metabolic disorders, treatment response is individual and varied. Many individuals remain at a high risk of developing CVD. Although genetic factors play a role in CVD pathogenesis, large-scale studies have revealed that they may only account for 20% of the risk of developing CVD (Ripatti et al., 2010). Thus, environmental factors play a dominant role in CVD pathogenesis.

Gut microbiota are an important environmental factor, and there is increasing evidence that the activity and composition of the gut microbiota are closely related to human diseases (Clemente et al., 2012; Sonnenburg and Backhed, 2016). It is believed that gut microbiota participate in the absorption of nutrients and energy, engage the innate immune system, and produce a wide variety of small-molecule metabolites that are sensed by host receptor systems to regulate host metabolism and inflammatory pathways relevant to CVD (Tang et al., 2017; Brown and Hazen, 2018). In the treatment of CVD, lowering risk factors such as hyperglycemia and hyperlipidemia, has been shown to reduce cardiovascular events (Grundy et al., 2004; Mazzone et al., 2008; Mooradian, 2009; Eliasson et al., 2011). In parallel, many metabolic disease treatments have also been shown to work through the gut microbiota. With the discovery of more microbial targets, an improved understanding of the specific effects of metabolic therapy drugs on gut microbiota is still required. This may also provide a new treatment strategy for the control of CVD risk factors.

In this review, we provide a brief overview on the contribution of the gut microbiota to metabolic disorders as well as CVD, and the response of gut microbiota to cardiovascular metabolic therapeutic drugs. In this manner, we link the existing studies on gut microbiota regulation by metabolic therapy drugs with their cardiovascular protective effects, suggesting that intestinal microorganisms may play a potential role in reducing cardiovascular risk factors. We also discuss the potential of microbiota-targeted therapeutics as novel treatment strategies for the preventing and/or treating CVD, and highlight the need to establish causal links between therapeutics for metabolic disease, gut microbiota modulation, and cardiovascular protection.

## The Role of Gut Microbiota Inmetabolic Disorders and Cardiovascular Disease

There are more than 1,000 microbial species in the human gastrointestinal tract, mainly belonging to five phyla (Bacteroidetes, Firmicutes, Actinobacteria, Proteobacteria, and Verrucomicrobia). Of these, anaerobic Bacteroidetes and Firmicutes contribute more than 90% of all bacterial species (Qin et al., 2010). They constitute 90% of the total number of cells contained within our bodies, and the number of microbial genes is over 100 times larger than the human nuclear genome (Grice and Segre, 2012; Hillman et al., 2017). Gene sequencing data have indicated that the gut metagenome is involved in host physiology and pathophysiology processes such as the digestion and absorption of nutrients, stimulation of the immune system, and regulation of host metabolic pathways (Costello et al., 2012; Nicholson et al., 2012; Palm et al., 2015). Harmful alterations in the gut microbiota composition, a condition referred to as dysbiosis, have been associated with the development of metabolic disorders. For instance, it is generally believed that a rise in the ratio of Firmicutes to Bacteroidetes is related to a chronic low-grade inflammatory status and an elevated capability for obtaining more energy from food (Pahwa et al., 2017). The diversity of gut microbiota in obese patients is visibly lower than that in the normal weight population (Wolf, 2006) and this decrease may lead to a higher insulin resistance (Jiao et al., 2018). In addition, there is a link between the gut microbiota and different kinds of CVD. The use of broad-spectrum antibiotics affects analytes produced during the catabolism of aromatic amino acids with an associated reduction in myocardial infarct size (Lam et al., 2016). A characteristic change in gut microbiota has been observed in coronary artery disease patients in which Lactobacillales increased and phylum Bacteroidetes (*Bacteroides* and *Prevotella*) decreased; this was not observed in a comparative cohort of patients with diabetes (Emoto et al., 2016). In another metagenome-wide association study, an increased abundance of *Enterobacteriaceae* and *Streptococcus* spp. and a relatively depleted abundance of butyrate-producing bacteria was observed in patients with atherosclerotic CVD compared to healthy controls (Jie et al., 2017).

Although the unified mechanism behind this kind of correlation has not yet been established, there are still some possible explanations for the interplay between gut microbiota and the host. In some cases, structural components of gut microbiota such as lipopolysaccharides (LPS) and peptidoglycans can be used as signaling molecules; these can be recognized as pattern recognition receptors, such as toll-like receptors (TLRs) and nucleotide oligomerization domain-containing receptors (Curtiss and Tobias, 2009; Philpott et al., 2014), which can stimulate and instruct host immune response both at the epithelial cell border as well as within the vasculature (Medzhitov, 2007). When gut wall barrier function is impaired, low levels of gut-derived bacteria can actually enter the bloodstream to elicit a chronic low grade pro-inflammatory and pro-oxidative stress status that is commonly referred to as “metabolic endotoxemia” which may result in several cardiovascular risk factors, such as obesity, insulin resistance, dyslipidemia, and oxidative stress (Neves et al., 2013).

Gut microbiota can also produce various metabolites that can act locally in the gut as well as travel systemically to directly or indirectly affect distal organs. Here, we provide a brief introduction to some main classes of gut microorganism-dependent metabolites that have been linked to CVD risk in either humans or mouse models.

Short-chain fatty acids (SCFAs) are products of the fermentation of dietary fibers by gut bacteria (Topping and Clifton, 2001). A growing body of evidence suggests that SCFAs, as metabolic targets, have a potential role in preventing and counteracting obesity and obesity-related diseases (impaired glucose metabolism and insulin resistance) (Canfora et al., 2015; Koh et al., 2016). By regulating the release of intestinal hormones, such as peptide YY and glucagon-like peptide-1 (GLP-1), SCFAs can suppress appetite, increase energy expenditure, and prevent diet-induced obesity (Gao et al., 2009; Lin et al., 2012; Chambers et al., 2015). Therefore, it may be possible to show that SCFAs are involved in cardiovascular physiological processes mainly by establishing links with cardiovascular risk factors (Canfora et al., 2015; Koh et al., 2016).

Bile acids (BAs) are an important group of metabolites with a profound effect on human health and microbiota are involved in the bio-transformation of BAs. For instance, bile acid deconjugation is carried out by bacteria with bile salt hydrolase (BSH) activity, this prevents the active absorption of BAs in small intestine via the apical sodium-dependent bile acid transporter (ASBT) (Wahlstrom et al., 2016). Besides, gut microbiota are the sole source of 7α and 7β dehydrogenase activity, which generates “secondary” bile acids (SBAs) such as deoxycholic acid (DCA) and lithocholic acid (LCA) (Wahlstrom et al., 2016). These bacterial modified bile acids regulate a wide range of metabolic and immune-related processes by acting as endocrine-like signaling molecules and engaging multiple bile acid receptors like farnesoid X receptor (FXR), pregnane X receptor (PXR), and G protein-coupled bile acid receptor (TGR5) (Wahlstrom et al., 2016; Joyce and Gahan, 2017; Brown and Hazen, 2018; Schramm, 2018). Importantly, studies have shown that the deficiency of either FXR or PXR in mice can cause reduced atherosclerosis, whereas TGR5 knockout mice are protected against atherosclerosis development (Zhang et al., 2006; Pols et al., 2011; Sui et al., 2011).

Trimethylamine oxide (TMAO) has gained considerable attention in recent years as a biomarker for human CVD risk and a promoter of atherothrombotic diseases (Wang et al., 2011; Zhu et al., 2016). After high-fat food intake, several distinct gut microbial enzyme complexes can metabolize nutrients such as phosphatidylcholine and choline to generate the primary gut microbial metabolite trimethylamine, which is subsequently converted by the host flavin monooxygenase enzyme family to TMAO in the liver (Bennett et al., 2013). TMAO then increases atherosclerotic CVD, including major adverse cardiovascular events (MACE) (death, myocardial infarction, and stroke) by altering cholesterol transport, modulating platelet hyperresponsiveness, and increasing macrophage activation (Tang et al., 2013; Koeth et al., 2014; Wang et al., 2015; Zhu et al., 2016).

In addition, bacterial metabolites originating from amino acids also play an important role in host physiology. For example, indole (a tryptophan metabolite) can be produced by many bacterial species including *Escherichia coli, Clostridium* spp., and *Bacteroides* spp. and has been shown to induce the release of GLP-1 in enteroendocrine L-cells. As signaling molecules, indole and some of its derivatives, such as indolepropionic acid (IPA) and indoleacrylic acid (IA) can affect mucosal homeostasis by decreasing intestinal permeability; this is possibly mediated by the PXR (Roager and Licht, 2018). Furthermore, IPA and IA also have anti-oxidative and anti-inflammatory effects (Karbownik et al., 2006; Wlodarska et al., 2017). A recent study also found that phenylacetylglutamine (PAGln), a new CVD-promoting gut microbiota-dependent metabolite originating from phenylalanine, increases the potential for thrombosis via G-protein-coupled receptors, leading to an increased risk of CVD and MACE (Nemet et al., 2020).

Collectively, both the structural components and metabolites of gut microbiota can be sensed by the host receptor system as signaling molecules, thus establishing direct or indirect interactions with the host. When dysbiosis occurs, many downstream signaling pathways are triggered, resulting in metabolic disorders, and indirectly or directly promoting CVD.

## The Effect of Oral Hypoglycemic Drugs on Gut Microbiota

### Metformin

As the first-line orally administered drug for T2DM treatment, metformin is one of the most prescribed compounds on the market, mainly due to its safety profile, proven efficacy, and low cost, as well as its high clinical value in reducing the incidence of cardiovascular events and mortality rates (UKPDS Group, 1998; Morgan et al., 2014). As far back as 1998, the UK Protective Diabetes Study (UKPDS) found that, compared to conventional treatment with diet alone, metformin treatment led to a lower all-cause mortality and a concurrent lower risk in the incidence of myocardial infarction in newly diagnosed T2DM after a median follow-up of 10.7 year (UKPDS Group, 1998). At the time, this phenomenon was explained as the beneficial effect of tight glycemic control from metformin that prevented future cardiovascular consequences. However, in a subsequent follow-up study 10 years after UKPDS, a continuous risk reduction of myocardial infarction was found in patients treated with metformin despite there being no changes in glycated hemoglobin (HbA1c) levels (Holman et al., 2008). Similar results have also been observed in subsequent clinical studies (Kooy et al., 2009; Hong et al., 2013; Han et al., 2019), which suggested the pleiotropic effects of metformin in the heart and blood vessels, independent of its glucose-lowering activity.

At present, the multiple action mechanisms of metformin are still debated, and the hypoglycemic effect has been primarily ascribed to its capacity to inhibit hepatic gluconeogenesis. However, despite the high accumulation of metformin in the intestinal wall, concentration in the plasma is up to 300 times lower (Bailey et al., 2008). In addition, intravenous administration of metformin does not improve glycemia (Bonora et al., 1984; Stepensky et al., 2002). This suggests that the intestine is possibly the main target organ of metformin action (Duca et al., 2015; Buse et al., 2016; Brunkwall and Orho-Melander, 2017). It is known that metformin can increase peripheral glucose uptake and modulate the incretin pathway by improving GLP-1 receptor expression in the pancreatic islets and increasing plasma levels of GLP-1 (Viollet et al., 2012; Pernicova and Korbonits, 2014; Montandon and Jornayvaz, 2017). GLP-1 is a major metabolic hormone produced by enteroendocrine L cells. Not only can GLP-1 lower blood glucose by inducing insulin secretion, inhibiting glucagon release, and slowing gastric emptying, it also instigates many cardioprotective pathways, possibly contributing to the cardiovascular protection observed in metformin users (Monami et al., 2017). The results of a human clinical study also provide consistent evidence for the intestine hypothesis. The delivery of delayed-release metformin with 50% of the bioavailability of extended-release metformin to the lower bowel results in significant glucose-lowering efficacy but with lower doses and significantly lower systemic exposure than that of extended-release metformin (Foretz et al., 2019). Although very little is currently known about the bacterial targets of metformin, there is convincing evidence that metformin can reshape the composition of human gut microbiota.

The abundance of *Akkermansia muciniphila*, a mucin-degarding bacterium, is reduced in individuals with obesity and diabetes compared to healthy individuals, as well as in rodents (Everard et al., 2013; Dao et al., 2016; Plovier et al., 2017). Furthermore, a higher abundance is associated with a healthier metabolic status and improvements in the cardiac metabolic parameters in individuals with obesity (Dao et al., 2016). Participants with diabetes that were also taking metformin had a higher relative abundance of this microbe compared to participants with diabetes that were not taking metformin and participants without diabetes (de la Cuesta-Zuluaga et al., 2017; Cani, 2018). This is in agreement with other *in vitro* experiments showing that metformin directedly increases the growth of *A. muciniphila* (Wu et al., 2017). As *Akkermansia* uses mucus as a nutrient source, Metformin is further found to increase the number of goblet cells. Goblet cells produce gastrointestinal mucins that protect the underlying epithelium from pathogens, and the number of it was positively correlated with the abundance of *Akkermansia* (Shin et al., 2014). In addition, one study found that a recombinant protein isolated by *A. muciniphila* can improve the intestinal barrier through interaction with TLR2 (Plovier et al., 2017), even A. *muciniphila* derived extracellular vesicles can influence gut permeability through the regulation of tight junctions (Chelakkot et al., 2018). Decreased gut permeability limits the release of endotoxins such as LPS, which in turn improves the chronic low-grade inflammatory status and reduces vascular inflammation (Tang et al., 2019). A recent study showed that fecal microbiota transplantation (FMT) using fecal material from metformin-treated mice can not only upregulate the expression of GLP-1 and pattern-recognition receptors TLR1 and TLR4 to improve hyperglycemia caused by a high-fat diet (HFD), but can also downregulate the expression of the inflammatory cytokine IL-18 (Lee et al., 2019) which has been identified as a possible risk factor for CVD (Jefferis et al., 2011). However, metformin treatment was recently found to be associated with an increased concentration of TMAO (a promoter of atherothrombotic disease) (Brown and Hazen, 2018; Croyal et al., 2020). Further, results from another animal study suggested that the plasma TMAO level were significantly associated with the relative abundance of *Akkermansia* within the jejunum and cecum (Koeth et al., 2014). Therefore, it is difficult to comprehensively determine whether increased *Akkermansia* abundance is ultimately beneficial for cardiovascular disease.

Another therapeutic effect of metformin is its influence on the interactions between BAs and the microbiota. The inhibitory effect of biguanides on bile acid absorption has been acknowledged since the 1970s (Caspary and Creutzfeldt, 1975). Metformin can reduce active bile acid absorption via ASBT inhibition (Napolitano et al., 2014). Subsequent modifications of bile acid-mediated activation of the nuclear FXR and the cell surface TGR5 might influence GLP-1 secretion from L cells (Bronden et al., 2017; van Stee et al., 2018). In a recent study, Sun et al. (2018) performed metagenomic and metabolomic analyses of samples from individuals with newly diagnosed T2DM after 3 days of metformin treatment. The BSH activity of *Bacteroides fragilis* was strongly reduced after metformin administration, and glycoursodeoxycholic acid (GUDCA) was increased in the gut. They further confirmed that GUDCA is an intestinal FXR antagonist that controls bile acid homeostasis and glycolipid metabolism (Matsubara et al., 2013). Thus, they concluded that metformin acts in part through a *B. fragilis* –GUDCA–intestinal FXR axis to improve metabolic dysfunction (Sun et al., 2018). In addition, *B. fragilis* is involved in the transformation of primary bile acids to SBAs such as LCA, which can activate the PXR (Staudinger et al., 2001). Considering the possible correlation between FXR, PXR and atherosclerosis (Zhang et al., 2006; Sui et al., 2011), the cardioprotective effect of metformin may be related to its regulation of *B. fragilis*. Moreover, levels of Firmicutes (*e.g., Clostridium perfringens*) have been shown to decrease in metformin-treated patients (Karlsson et al., 2013), which in turn can alter bile acid metabolism by reducing the circulating levels of pro-inflammatory bile acids (DCA and TDCA) (Fujisaka et al., 2018).

Metformin can also increase the abundance of SCFA-producing taxa and species (e.g., *Butyrivibrio, Bifidobacterium bifidum*, and *Megasphaera*) (Forslund et al., 2015; de la Cuesta-Zuluaga et al., 2017). As mentioned earlier, SCFAs function as a macronutrient energy source and hormone-like signaling molecules that enter the portal circulation to signal through two orphan G protein-coupled receptors, GPR41 (Samuel et al., 2008) and GPR43 (Kimura et al., 2013), and mediated AMPK activation in diverse tissues to regulate innate immunity and host metabolism (den Besten et al., 2015; Brown and Hazen, 2018). In addition, metformin can increase the enrichment of *Lactobacillus* and *Bifidobacterium* (Wu et al., 2017). Whereas *Lactobacillius* promote GLP-1 release from L-cells by increasing the apical expression of sodium glucose cotransporter-1 (SGLT1) (Bauer et al., 2018), *Bifidobacterium* have also proven to be negatively related to HbA1c.

Although the intestine hypothesis of the metformin action mechanism provides an explanation for its cardiovascular benefits, this still requires further validation, especially regarding the role of metformin in non-diabetic patients with CVD. Limited by indications, there has been very limited research on this subject. A small randomized double-blind placebo-controlled study consisting of 33 non-diabetic women showed that metformin can improve vascular function and reduce myocardial ischemia in female patients with angina compared to a placebo (Jadhav et al., 2006). In addition, the recent proof-of-concept MET-REMODEL trial (Metformin and its Effects on Left Ventricular Hypertrophy in Normotensive Patients With Coronary Artery Disease) in patients with coronary artery disease (CAD), insulin resistance (IR), and/or pre-diabetes found a beneficial effect of metformin on left ventricular mass indexed to height (LVMI), LVM, office systolic blood pressure, and oxidative stress (Mohan et al., 2019). However, some pilot clinical studies with other surrogate end-points for cardiovascular events such as carotid intimal media thickness have not yielded positive results (Preiss et al., 2014; Kulkarni et al., 2018; Rena and Lang, 2018). Thus, further evidence is needed before metformin can be recommended for non-diabetic patients with CVD.

### Alpha- Glucosidase Inhibitors

Accumulating evidence suggests that post-prandial hyperglycemia (PPHG) is a powerful predictor of diabetic cardiovascular events (DECODE Study Group, 2001; Ning et al., 2010). Studies have found that PPHG can increase oxidative stress through endothelial dysfunction (Kawano et al., 1999) and low-grade chronic inflammation (Esposito et al., 2002), leading to the development of atherosclerosis and the occurrence of cardiovascular events (Node and Inoue, 2009). Alpha-glucosidase inhibitors (α-GIs) are microorganism-derived anti-glycemic drugs that have been proven to reduce PPHG and are associated with a favorable impact on an array of CVD surrogate markers (Geng et al., 2011; Standl et al., 2014).

The classic mechanism of α-GIs is believed to act through the inhibition of the host glucoamylase, delaying carbohydrate digestion and pushing the carbohydrates to the lower intestinal tract, where they can be transformed into the food for the intestinal bacterial community. This makes it possible to change the gut microbiota composition. Although the specific mechanism of α-GIs that regulates the gut microbiota is unclear, there is still evidence that apart from reducing post-prandial blood glucose, AGIs can also protect the cardiovascular system by reshaping the gut microbiota composition.

Acarbose, which is derived from the fermentation process of *Actinoplanes utahensis*, is the most widely used α-GI in hyperglycemia treatment (Weng et al., 2015). Previous studies suggest that acarbose treatment can reduce the risk of cardiovascular endpoints in patients with diabetes (Hanefeld et al., 2004; Hanefeld, 2007; Chen et al., 2014). A clinical study involving 106 patients with newly diagnosed T2DM found that acarbose treatment can significantly change the gut microbiota composition compared to glipizide, especially increasing the abundance of species possessing high BSH activity, such as *Lactobacillus gasseri* and *Bifidobacterium longum*, while depleting putrefactive species of *Bacteroides, Alistipes* and *Clostridium*, thereby changing the relative abundance of microbial genes involved in BA metabolism and contributing to beneficial effects on the host metabolism (Gu et al., 2017). Another study confirmed this result (Su et al., 2015), which also found that acarbose can reduce the level of LPS in the blood by increasing the number of *Bifidobacterium* and reducing the number of *Enterococcus faecalis* (Kikuchi et al., 2018), and thus, alleviate chronic low-level inflammation in patients. These results indicate that changes in the gut microbiota after acarbose treatment can bring greater cardiovascular benefits to diabetic patients. In addition, the cardiovascular benefits of acarbose can be explained by new insights into the production of SCFAs and the release of H_2_ gas by the gut microbiota. Increasing evidence supports the view that the use of acarbose increases the number of colonic bacteria, which in turn promotes the production of SCFAs and H_2_ gas through the fermentation of undigested carbohydrates in the lower digestive tract (Suzuki et al., 2009; Hai-xia et al., 2011). As mentioned previously, SCFAs can signal through specialized host receptor systems to regulate innate immunity and host metabolism. H_2_ gas can be absorbed from the intestine into the circulation to mediate the inhibition of pro-inflammatory cytokines, especially IL-1β, TNF-α, and IL-6 in inflammatory tissues. Moreover, a recent study proposed that H_2_ converts the quinone intermediates into the fully reduced ubiquinol, thereby increasing the antioxidant capacity of the quinone pool and preventing the generation of reactive oxygen species (Ishibashi, 2019). Clinical evidence and experimental results strongly suggest that reactive oxygen species are the main cause of cardiovascular diseases such as hypertension, atherosclerosis, angina pectoris, myocardial infarction, and heart failure (Touyz, 2004; Madamanchi et al., 2005).

Although current microbiota studies support acarbose for its cardiovascular benefits, its cardioprotective effect in non-diabetic patients is still controversial. The STOP-NIDDM trial (Study to Prevent Non-Insulin-Dependent Diabetes Mellitus) showed that decreasing PPHG with acarbose was not only associated with a reduction in the incidence of new-onset diabetes as the primary objective of this study, but also with a relative risk reduction in the development of cardiovascular events, especially in the risk of myocardial infarction as assessed by a planned *post hoc* analysis of predefined secondary outcomes (Chiasson et al., 2003). However, the recently completed ACE (Acarbose Cardiovascular Evaluation) trial on patients with coronary heart disease and impaired glucose tolerance showed that acarbose delayed progression to type 2 diabetes, but was neutral with respect to MACE (Holman et al., 2017).

### GLP-1 Receptor Agonists

As mentioned earlier, GLP-1 is an insulinotropic hormone secreted by gastrointestinal neuroendocrine cells. The body can help to reduce blood sugar by secreting GLP-1 after eating, but natural GLP-1 is rapidly degraded by DPP-4 secreted in the intestine. Due to the relative lack of insulin or insulin resistance, diabetes patients have high blood glucose levels. At this time, auxiliary substances such as GLP-1 are particularly important. GLP-1 receptor agonists (GLP-1RAs), a new type of hypoglycemic drug, can not only increase insulin secretion, suppress appetite, and control weight by activating GLP-1 receptors (Vilsboll et al., 2012; McAdam-Marx et al., 2014), they can also control hyperglycemia by inhibiting glucagon secreted by islet α cells (Meier, 2012). Of these, liraglutide (Marso et al., 2016b) and semaglutide (Marso et al., 2016a) have also been found to have cardioprotective effects. Interestingly, although all GLP-1RAs currently on the market are subcutaneous injections, liraglutide can change the composition of microorganism community (Zhang et al., 2018; Madsen et al., 2019). This may be due to the GLP-1 level because it influences the gut transit time and gastric emptying rate, and could therefore modify the gut lumen internal environment (local pH value and nutrient composition).

Zhang et al. (2018) found that SCFA-producing bacteria including *Bacteroides, Lachnospiraceae*, and probiotic *Bifidobacterium*, arec selectively enhanced in liraglutide-treated diabetic male rats. SCFA can affect intestinal anti-inflammatory abilities (Boulange et al., 2016) and prevent a low-grade inflammatory response. In addition, *Lachnospiraceae* exhibit a positive relationship with peak oxygen uptake, the gold standard measure of cardiorespiratory fitness (Estaki et al., 2016). Fuerthermore, liraglutide can reduce obesity-associated bacteria (*Erysipelotrichaceae, Marvinbryantia, Roseburia, Candidatus* Arthromitus, *Parabacteroides*, Romboutsia, and Ruminiclostridium) in both obese and diabetic obese rats (Wang et al., 2016; Zhao et al., 2018) and increased the lean-related phylotypes such as Prevotella, Blautia and Coprococcus. The *Akkermansia* genus was significantly reduced in patients with a long T2DM duration. After comparing the gut microbiota of subjects receiving GLP-1 RA and metformin, a higher *Akkermansia* abundance was found in the liraglutide-treated patients (Wang et al., 2018b). As mentioned earlier, a higher abundance of *Akkermansia* is beneficial for metabolism and the gut barrier function, it is also related to the plasma concentration of TMAO. However, it is still unknown whether liraglutide treatment can result in increased TMAO concentrations.

It has been found that GLP-1 RA can reduce the formation of atherosclerosis by improving vascular endothelial function, inhibiting inflammatory response, and reducing myocardial infarction ischemia, thereby preventing diabetic vascular disease (Khat and Husain, 2018). Although there are few pilot clinical studies on using GLP-1RA to prevent CVD, the regulation of gut microbiota may also become one of the potential cardiovascular benefit mechanisms of GLP-1RA in the future.

### Sodium Glucose Co-transporter 2 Inhibitors (SGLT2i)

SGLT2i are a new class of oral hypoglycemic drugs. The action mechanism of SGLT2i mainly promotes urine glucose excretion by inhibiting the reabsorption of sodium glucose in the proximal renal tubules. Due to its unique glucose excretion mechanism, the hypoglycemic effect of SGLT2 inhibitors is unaffected by the function of islet cells and insulin resistance, thus reducing the risk of hypoglycemia (Whalen et al., 2015). Recently, as the benefits of SGLT-2i in cardiovascular outcome trials (COVTs) outweigh those of other anti-diabetic drugs (Zinman et al., 2016; Neal et al., 2017; Wiviott et al., 2019), they have received increasing attention in the field of diabetes. Three CVOTs on empagliflozin, canagliflozin, and dapagliflozin in T2DM patients and either established CVD or multiple cardiovascular risk factors showed remarkable positive results and proved the significant role of these three types of SGLT2i in reducing MACE, cardiovascular mortality, all-cause mortality, and hospitalization for heart failure (Zinman et al., 2016; Neal et al., 2017; Wiviott et al., 2019).

Based on our current understanding, the cardiovascular protection mechanism of SGLT2i mainly lies in their ability to reduce cardiac inflammation, oxidative stress, cell apoptosis, mitochondrial dysfunction, and ionic abnormalities (Lahnwong et al., 2018). As of yet, consistent evidence for their effect on microbiota is lacking. A pharmacological experimental study of a dual SGLT1/2 inhibitor complex showed that the relative abundance of both the bacterial orders and bacteria of interest in metabolic disease (e.g., *Akkermansia* spp.) in C57BL/6 mice was unaltered after drug treatment (1 mg/kg p.o. for 6 consecutive days) (Lahnwong et al., 2018). Another animal experiment for dapagliflozin (dapa) (Lee et al., 2018) found that treatment with dapa had little effect on the gut microbota of control mice, but did subtly change the richness and diversity of the microbial community in diabetic Db mice. On the one hand, the Firmicutes to Bacteroidetes ratio in the Db + dapa group was reduced, which suggests that dapa may reduce the level of inflammation in the body by adjusting the gut microbiota. The experiment also found that at the species level, *A. muciniphila* showed a tendency to increase in the Db + dapa group compared to the Db group. *A. muciniphila* has been shown to improve metabolic outcomes, including vascular function (Qin et al., 2012; Li et al., 2016), while participating in the protection of the intestinal mucosal barrier and controlling low-grade inflammation (Chelakkot et al., 2018; Lee et al., 2019; Tang et al., 2019).

### Dipeptidyl Peptidase-4 Inhibitors (DPP-4i)

DPP-4i are an extensively used class of oral hypoglycemic agents that target the DPP-4 enzyme and inhibit the degradation of GLP-1 to reduce blood glucose levels (Holst and Deacon, 1998). Although the existing CVOTs with DPP-4i showed no difference in combined MACE outcomes (Scirica et al., 2013; White et al., 2013; Green et al., 2015), DPP-4i may also positively influence surrogate vascular end points and other cardiovascular risk factors, as extensively discussed in previous reviews (Mulvihill and Drucker, 2014; Scheen, 2018). In parallel, some progress has been made in identifying the influence of DPP-4i on gut microbiota, although most of the evidence comes from animal experiments.

Currently, DPP-4i studies, especially those on sitagliptin and vildagliptin, have shown similar evidence that DPP-4i treatment partially reverses HFD-induced dysbiosis, significantly reduces the Firmicutes to Bacteroidetes ratio, and alters the population of SCFA-producing bacteria. Vildagliptin also has a potential effect on inflammation due to the reduction of TLR and cytokine expression (Yan et al., 2016; Zhang et al., 2017). All these changes ultimately mediate the beneficial effects of DPP-4i on the host, especially in terms of glucose homeostasis. Another animal study found that both sitagliptin and saxagliptin alter gut microbial composition and promote a functional shift in the gut microbiome. In particular, succinate production is especially increased. Succinate is reportedly a key substrate of intestinal gluconeogenesis in the improvement of glucose metabolism (de Vadder et al., 2016). And GLP-1 is not the main mediator of the effect on gut microbiota, a suggestion that is further supported by the findings of Olivares et al., who proposed the existence of significant intrinsic DPP-4-like activity in gut microbial populations (Olivares et al., 2018). Moreover, by transferring fecal samples from sitagliptin-treated T2DM patients to HFD-fed germ-free mice, this study further demonstrated that the altered microbiome contributes to the hypoglycemic effects of sitagliptin, even in the absence of additional treatments (Liao et al., 2019).

## The Effect of Statin on Gut Microbiota

Hyperlipidemia is an important risk factor for CVD (Isomaa et al., 2001) and is one of the main results of multiple metabolic disorders. Statins, as the most widely used drugs for treating hyperlipidemia, have been recommended by the American College of Cardiology/American Heart Association (ACC/AHA) as first-line treatment for hyperlipidemia (Stone et al., 2014). Lipid metabolism regulation is mainly achieved by inhibiting 3-hydroxy-3-methylglutaryl coenzyme-A reductase in the cholesterol synthesis pathway (Stancu and Sima, 2001). Although the efficacy of statins has been proven, their therapeutic effects vary from person to person. A meta-analysis of a statin intervention trial involving 32,258 patients in 37 clinical trials showed that the standard deviation of low-density lipoprotein cholesterol (LDL-C) reduction for all statins and doses ranged from 12.8 to 17.9% (Karlson et al., 2016). The percentage of patients experiencing a suboptimal response (<30% reduction in LDL-C) ranged from 5.3 to 53.3%. The change was not related to the specific statin dose, but was probably related to the composition of the gut microbiota. In an observational study involving 64 patients with hyperlipidemia (Liu et al., 2018), after 4–8 weeks of rosuvastatin treatment, there was a difference in the microbiota composition of the two groups that showed the best (group I) or suboptimal (group II) clinical results. In particular, the higher abundance of Firmicutes, butyrate-producing bacteria families of *Ruminococcaceae, Lachnospiraceae*, and *Clostridiaceae*, and the lower abundance of Bacteroidetes are believed to be related to the best therapeutic effect of rosuvastatin. However, considering the fact that the study lacked a control group and did not sequence the bacteria in either group before treatment, microbiome changes could not be assessed. In another experiment with atorvastatin in rats (Khan et al., 2018a), atorvastatin-treated HFD groups showed a relative increase in biodiversity compared to the HFD control group, and promoted the relative abundance of Proteobacteria, and reduced the abundance of Firmicutes. At the same time, several specific dominant groups including *Oscillospira, Parabacteroides, Ruminococcus*, unclassified CF231, YRC22 (*Paraprevotellaceae*), and SMB53 (*Clostridiaceae*) were observed to have a reversed abundance relative to the HFD group, which was similar to that of the normal rat food control group. In a cross-sectional study, the gut microbiome of 15 untreated hypercholesterolemia patients, 27 atorvastatin-treated hypercholesterolemia patients, with 19 healthy subjects were compared (Khan et al., 2018b). A distinct bacterial signature with species associated with atherosclerosis and inflammation (e.g., *Collinsella, Streptococcus*) was found in the untreated hypercholesterolemia patients, while an increased abundance of putative anti-inflammatory species (*Akermansia muciniphila, Faecalibacterium prausnitzii*, and genus *Oscillospira*) was found in atorvastatin-treated patients.

**Figure 1:**
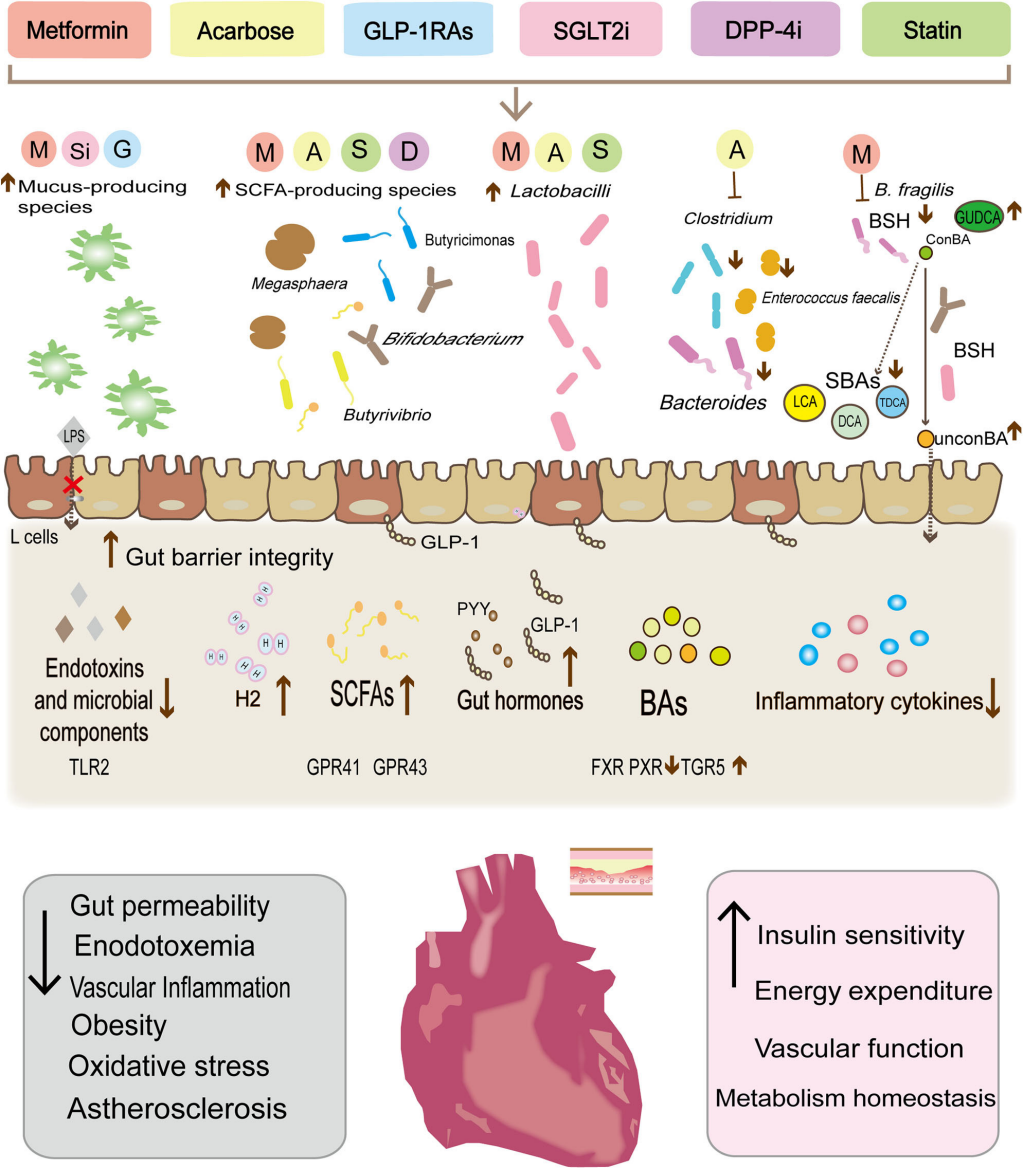
Metabolic therapy drugs reshape the gut microbiota composition, modulate the abundance of specific gut bacteria, and then control the release of the microbial derivative to achieve cardiovascular protection and reduce cardiovascular risk factors. Metformin, liraglutide, and dapalizine have been found to increase the abundance of *Akkermansia muciniphil*, which can enhance the function of the intestinal barrier, limit endotoxin leakage. In parallel, the increase in the abundance of some species (Bacteroides, Butyricimonas, Lactobacilli, and Mucispirillum) after drug treatment has also been found to be significantly related to the decrease in secretion of pro-inflammatory cytokines (IL-18, IL-1β, IL-16, and TNFα). In turn, the decrease of the low-level inflammatory status has been shown to increase insulin sensitivity and benefit the vascular function. Similarly, both metformin and acarbose can reduce *Bacteroides* and *Clostridium* clusters, and may alter bile acid metabolism, with a reduction of bile acid reabsorption and secondary bile acids, thereby influencing the excitability of various atherosclerosis-related receptors (PXR, FXR, and TGR5) and stimulating GLP-1 secretion. Moreover, metformin, acarbose, liraglutide, rosuvastatin, and atorvastatin have also been found to increase SCFAs-producing species (*Butyrivibrio, Bifidobacterium, Megaspheara, and Butyricimonas Lactobacilli* et al.). SCFAs, through the G protein-coupled receptors GPR-41 and GPR43 mediated AMPK activation in diverse tissues to regulate innate immunity and host metabolism. In addition, *Lactobacilli* can also promote GLP-1 secretion by increasing the apical expression of SGLT1 cotransporter. Finally, acarbose increases the release of H_2_ gas by the gut microbiota as well as preventing oxygenated stress damage.

To investigate the role of gut microbiota in the effect of statins on lowering blood lipid, Wang et al. (2018a) used antibiotics to establish a rat model of microbiome imbalance that lasted for 2 weeks. They found that the abundance of *Lactobacillus* and *Bifidobacterium* was remarkably diminished upon antibiotic treatment in the antibiotic+rosuvastatin-treated group compared to that of the rosuvastatin-treated group and control group. Correspondingly, the efficacy of rosuvastatin in lowering the blood levels of total cholesterol and LDL-C was significantly compromised. Studies have shown that the presence of *Bifidobacterium* and *Lactobacillus* can lead to the alteration of TNFα and IL-6 levels, which results in the reduction of cholesterol (Veiga et al., 2005). The expression of a cellular transporter of rosuvastatin (OATP1B1) is regulated by a variety of factors including TNFα and IL6 (Le Vee et al., 2009); therefore, it can be speculated that beneficial gut bacteria mediate the lipid-lowering effect of rosuvastatin by influencing the expression of inflammatory factors. In a recent study, Kim et al. (2019) found that atorvastatin and rosuvastatin significantly increased the abundance of the genera Bacteroides, Butyricimonas, and Mucispirillum in elderly obese mice. This change in gut microbiota plays an important role in down-regulating IL-1β expression and upregulating TGFβ expression (Winer et al., 2016; Goncalves et al., 2018) (Figure 1).

In addition to the impact of statin on gut microbiota, there is also evidence that the efficacy of statin medications is regulated by gut microbiota. A study involving plasma metabolomic profiling in 100 human subjects found that baseline concentrations of three secondary bacterial-derived BAs (LCA, taurolithocholic acid, and glycolithocholic acid), positively predicted the magnitude of simvastatin-induced LDL-C lowering (Kaddurah-Daouk et al., 2011). An animal study also reported that simvastatin treatment increased the abundance of Bacteroidetes, reduced Firmicutes and Actinobacteria, as well as altering the serum metabolic and bile acids profiles of HFD feeding mice. Moreover, it found that gut microbiota modulation resulting from oral antibiotics attenuated the hypolipidemic effect of simvastatin and suggested that this might be related to the suppression on hepatic CYP7A1, CYP7B1, and FXR proteins that regulate bile acids synthesis from cholesterol by gut microbiota modulation (He et al., 2017).

## Discussion

With the development of gene sequencing technology and human microbiome research, an increasing amount of evidence from animal and human studies has revealed that the activity of gut microbiota is closely related to the occurrence and development of metabolic and CVD. In parallel, gut microbiota have been recognized as playing an important role in the digestion, absorption, metabolism and even the effect of drugs. Therefore, with an in-depth understanding of gut microbiota, it is possible that microbial pharmacology may become an important part of modern pharmacology.

In this review, we summarize the advances of research on several drugs for metabolic diseases with proven cardiovascular benefits in regulating gut microbiota. This enables the identification of microbial targets that may help these drugs to provide their cardiovascular protective effects. At present, the potential benefits of these drugs in reducing CVD risks mainly include the reduction of multiple cardiovascular risk factors such as hyperglycemia, hypertension and hyperlipidemia, as well as other beneficial effects, including increased insulin sensitivity, weight loss, reduced of oxidative stress and low-grade inflammation, restored of endothelial function, and the inhibition of key platelet activation pathways (Standl et al., 2014; Boyle et al., 2018; Lahnwong et al., 2018; Zilov et al., 2019). The effects of these drugs on the gut microbiota could be one explanation for most of these benefits.

Despite some exciting findings, few studies provide causal evidence that drugs are directly involved in cardiovascular protection through the gut microbiota. On the one hand, gut microbiota vary greatly from individual to individual. The genetic background, lifestyle, emotional state, and method of childbirth of the host all have a great influence on gut microbiota composition (Wen and Duffy, 2017). On the other hand, gut microbiota are not homogenous along the digestive tract, especially between the mucosa (cecum or colon) and stool (Eckburg et al., 2005). Most of the existing studies have selected samples from the cecum, colon, and feces for gene sequencing, and the results are often offset by the location of the selected tissues. Therefore, more study designs using antibiotics or FMT should be encouraged to clarify the causal relationship between microbiome changes and CVD, then to verify these findings through large-sample prospective clinical trials.

Among the drugs we have discussed, there is no doubt that metformin is the most studied. Studies in rodents and humans have generally suggested that metformin can protect the cardiovascular system by increasing SCFA-producing bacteria, interfering with the bioconversion of BAs, and promoting the growth of anti-inflammatory probiotics. Even if the increased TMAO concentration associated with metformin has a negative effect on the cardiovascular system, one may hypothesize that considering the overall effects on the gut microbiota, metfomin treatment could still result in positive cardiovascular outcomes. However, there are still few studies on other drugs mentioned in this manuscript. Current researches speculates that they may achieve regulate immunity, host metabolism, and oxidative stress by promoting the growth of beneficial bacteria and inhibiting the expansion of harmful bacteria, thereby indirectly providing cardiovascular protection. However, many studies have analyzed the gut microbiota at the phylum level. Expressions such as “SCFA-producing bacteria” and “Firmicutes to Bacteroidetes ratio” may be too simple and broad, because these bacteria are diverse, widely distributed and even compete with one another (Becker et al., 2011). There have been many attempts at treatment, such as probiotic supplementation and FMT (Brown and Hazen, 2017), we believe that with the reduction of sequencing costs, metagenomic sequencing will become more and more popular, and the refinement of microbiome structure analysis will help people better understand, screen and utilize the specific functions of microbiota.

In addition, research on the molecular biological mechanisms of drug-microbe interactions and microbe-host interactions are still in progress. Existing studies show that microbial derivatives play an important role in regulating human physiological activities and protecting the cardiovascular system. These derivatives might include new mediators and potential pharmacological targets for the treatment of cardiometabolic diseases. Non-lethal small-molecule inhibitors of TMAO production can protect mice from diet-enhanced atherosclerosis (Wang et al., 2015). Therefore, additional molecular biology studies on both drug-microbe interactions and microbe-host interactions are still needed. The amount of chemicals and metabolites derived by gut microbiota is staggering. Once a desired microbial derivative is identified, it may become a new drug target for cardiometabolic disease.

## Conclusion

The current microbiome studies on antidiabetic agents and lipid lowering agents mainly focus on the role of gut microbiota in regulating metabolic disorders. However, several metabolic therapy drugs have been proven to have additional cardiovascular benefits. Simultaneously, the influence of gut microbiota on the cardiovascular system is being recognized gradually. In this review, we linked the recent advances in microbiome studies on metabolic therapy drugs with their cardiovascular protective effects. Endogenous or exogenous factors change gut microbiota composition, which affect human physiological functions by releasing and regulating a variety of mediators including SBAs, TMAO, SCFAs, inflammatory factors, endotoxins, and intestinal hormones. Existing metabolic therapy drugs may regulate metabolism, immunity, oxidation-reduction balance, and other functions by changing the abundance of certain bacteria in the intestinal tract, thus providing cardiovascular protection. However, more detailed experimental and clinical investigations are needed in order to better understand the molecular mechanism of these drugs' regulation of gut microbiota and to identify new targets for microorganism-targeted therapeutics.

## Author Contributions

Q-YD and J-XT conceived the work. Q-YD prepared the first draft of the manuscript and designed the figure. X-lT, L-HZ, ML, and F-ML critically revised the manuscript and agreed with the manuscript's results and conclusion. X-XW, LH, Y-JZ, Z-ZG, H-YY, and X-YF contributed to the writing of the manuscript. All authors critically reviewed and approved the final version of the article.

## Conflict of Interest

The authors declare that the research was conducted in the absence of any commercial or financial relationships that could be construed as a potential conflict of interest.
